# Melatonin promotes the BMP9-induced osteogenic differentiation of mesenchymal stem cells by activating the AMPK/β-catenin signalling pathway

**DOI:** 10.1186/s13287-019-1511-7

**Published:** 2019-12-21

**Authors:** Tianyuan Jiang, Chao Xia, Xiaoting Chen, Yan Hu, Yan Wang, Jin Wu, Shuyan Chen, Yanhong Gao

**Affiliations:** 1 Department of Geriatrics, Xinhua Hospital, Shanghai Jiaotong University School of Medicine, Shanghai, 200092 China; 2 Shanghai Institute for Pediatric Research, Shanghai Jiaotong University School of Medicine, Shanghai, 200092 China

**Keywords:** Melatonin, BMP9, Osteogenic differentiation, Mesenchymal stem cells

## Abstract

**Background:**

Mesenchymal stem cells (MSCs) play a crucial role in maintaining the dynamic balance of bone metabolism. Melatonin may have a regulatory effect on bone metabolism by regulating the lineage commitment and differentiation signalling pathways of MSCs. Among the BMP families, the osteogenesis of BMP9 is considered to be one of the strongest in MSCs. Here, we explored whether melatonin and BMP9 act synergistically on MSC osteogenic differentiation.

**Methods:**

The C3H10T1/2 osteogenic differentiation function induced by melatonin synergizes with BMP9, as detected by the expression of osteogenic markers at different periods. The result was further confirmed by foetal limb explant culture and in vivo stem cell implantation experiments. The effects of the AMPK/β-catenin pathway on the osteogenic differentiation of C3H10T1/2 cells were evaluated by Western blotting.

**Results:**

Melatonin combined with BMP9 significantly enhanced the expression of osteogenic markers at different periods in C3H10T1/2 cells, effectively enhancing BMP9-induced bone formation in cultured foetal explants and ectopic bone formation in vivo in stem cell transplantation experiments. Melatonin increases the expression of BMP9 in C3H10T1/2 cells and induces Smad1/5/8 translocation from the cytoplasm to the nucleus. In addition, melatonin and BMP9 synergistically promote AMPK and β-catenin phosphorylation, which can be largely eliminated by AMPK siRNA pretreatment.

**Conclusions:**

Melatonin and BMP9 in C3H10T1/2 cells synergistically promote osteogenic differentiation at least in part by activating the AMPK/β-catenin signalling pathway.

## Background

Mesenchymal stem cells (MSCs) are pluripotent stem cells derived from mesoderm that can differentiate into osteoblasts, adipocytes and chondrocytes with strong self-renewal and multi-directional differentiation [1, 2]. Bone is one of the few organs that retains the potential for regeneration and can be continuously remodelled throughout life [3]. The osteogenic differentiation of MSCs plays an important role in bone regeneration and remodelling. This process is regulated by a variety of hormones, transcription factors and cellular signalling pathways, including bone morphogenetic protein (BMP), Wnt, insulin-like growth factor (IGF), epidermal growth factor and growth hormone, and is involved in multiple pathway interactions [3–6].

Bone morphogenetic proteins (BMPs) are important signalling pathways for cell proliferation and differentiation during embryonic development and play a key role in regulating the osteogenic differentiation of mesenchymal stem cells [7]. BMPs belong to the transforming growth factor (TGF) superfamily and have at least 15 different subtypes present in humans. BMP2, BMP4, BMP7 and BMP9, all play an important role in promoting osteogenic differentiation and bone formation. Among these proteins, BMP2 and BMP7 have been evaluated in clinical trials for the treatment of tibial fractures and spinal fusion [8, 9]. BMP9 is considered to be one of the most effective factors for inducing mesenchymal stem cell osteogenesis in various BMPs. BMP9 phosphorylates the transcription factor smad1, smad5 or smad8. These phosphorylated r-smad factors bind to smad4 and localize to the nucleus to promote the expression of osteogenic genes [10, 11]. In addition, some factors or signals, such as Wnt/β-catenin, ATRA and EGF, interact with BMP9 to enhance the BMP9-induced osteogenic differentiation of MSCs [3, 4, 12].

Melatonin (N-acetyl-5-methoxytryptamine) is a hormone secreted by the pineal gland that affects circadian rhythm, regulates the sleep-wake cycle, inhibits tumour growth and regulates immunity [13]. In most mammals, including humans, melatonin receptors have two subtypes, MT1 and MT2, which belong to the G protein-coupled receptor family [14, 15]. Melatonin exerts its physiological regulation mainly through receptors distributed in the hypothalamus (PT) and suprachiasmatic (SCN), and peripheral tissues, such as the nervous system, retina, immunity system, reproductive system and endocrine system tissues, also show a distribution of these receptors. Studies have shown that melatonin may have a regulatory effect on bone metabolism: promoting the osteogenic differentiation of BMSCs while inhibiting adipogenic differentiation [16, 17]; inhibiting osteoclast formation and activation, thereby inhibiting bone resorption [18]; and activating the antioxidant defence system to maintain the self-renewal and differentiation of BMSCs after long-term passage [19]. Additionally, the expression of melatonin receptors was also found in MSCs. It is currently believed that the osteogenic effects of melatonin are mainly mediated through the MT2 receptor, and the mechanisms involved include TGF-β [20], the Wnt/β-catenin pathway [15] and the MAPK signalling pathway [21]. It has been found that in pituitary AtT20 cells and rat granulosa cells, melatonin promotes the expression of BMP4 or BMP6 and phosphorylation of Smad1/5/8 downstream of BMPs [22, 23]. The melatonin-induced expression of osteogenic markers, such as BMP4 or BMP2, was also found in mouse pre-myoblast cell lines, C2C12 cells and human dental pulp stem cells [24, 25]. However, whether this effect of melatonin on BMPs is also present in MSCs and how the interaction between melatonin and BMPs occurs during the osteogenic differentiation of MSCs is still unclear, so further research is needed.

The specific purpose of this study was to investigate the effects of melatonin on the BMP9-induced osteogenic differentiation of MSCs and to reveal the underlying molecular mechanisms of this effect. Our results suggest that melatonin combined with BMP9 promotes osteogenic differentiation by activating the AMPK/β-catenin signalling pathway. Therefore, melatonin may provide another potentially effective option for enhancing BMP9-induced osteogenesis in bone marrow mesenchymal stem cells. This effect has important clinical significance for promoting the application of MSCs in the field of bone tissue engineering and research, treatment and prevention of the pathogenesis of bone metabolism diseases, such as osteoporosis, and fracture healing.

## Methods

### Cell culture and chemicals

C3H10T1/2 cells were purchased from Shanghai Institutes for Biological Sciences (Shanghai, China), and the cells were cultured in Dulbecco’s modified Eagle’s medium (DMEM) with 10% foetal bovine serum (FBS), 100 U/ml penicillin and 100 mg/ml streptomycin. The culture environment was 37 °C in 5% CO^2^. Melatonin was diluted to 100 mM as storage concentrations in DMSO. DMSO was used as a solvent control. Unless otherwise indicated, all chemicals were purchased from Sigma-Aldrich (St. Louis, MO, USA).

### Alkaline phosphatase (ALP) activity and staining

The total cellular protein concentration of the sample was determined using a BCA protein assay (Beyotime, China). The activity of ALP was quantitatively determined by modified Great EscAPe SEAP Chemiluminescence Assay (BD Clontech, USA) as previously described. According to the definition of enzyme activity, the alkaline phosphatase activity in the sample was calculated. Qualitative detection of ALP activity was performed using a BCIP/NBT alkaline phosphatase staining assay kit (Beyotime, China).

### Alizarin red S staining

Alizarin red S staining was carried out as described above [8–10]. After the corresponding treatment, C3H10T1/2 cells were cultured in the presence of 50 μg/ml ascorbic acid, 100 nM dexamethasone and 10 mM β-glycerophosphate. The medium was changed every 3 days. After 21 days of culture, the medium was removed, the cells were washed twice with PBS and fixed at room temperature for 15 min using 4% paraformaldehyde, and the cells were washed twice more with PBS. The fixed cells were incubated with 2% Alizarin red S solution (Sigma-Aldrich) for 10 min at room temperature and then washed thoroughly in PBS. Calcium mineral deposition staining was observed by light microscopy (Leica DMI 3000B, Germany).

### Immunohistochemical (IHC) staining

After 14 days of the corresponding treatment, C3H10T1/2 cells were fixed with 4% paraformaldehyde and washed with PBS. The cells were permeabilized with 0.1% Triton-X (Sigma, USA) and blocked with 10% BSA (Beyotime, China) to reduce non-specific staining. Subsequently, the cells were incubated with anti-osteocalcin (OCN) antibody (sc30045, Santa Cruz Biotechnology) and anti-osteopontin (OPN) antibody (ab91655, Abcam) at 4 °C overnight. The cells were washed three times with DPBS and incubated with a biotin-labelled secondary antibody (Santa Cruz Biotechnology, USA) for 20 min at 37 °C. The cells were washed three times with DPBS, and a streptavidin-HRP conjugate was added to the cells to incubate for 20 min at 37 °C. The presence of the expected protein was observed by diaminobenzidine (DAB) staining and examined under a microscope. Control IgG staining was used as a negative control.

### Western blot analysis

The cells were lysed by ice-cold RIPA lysis buffer (Biyuntian, China) containing protease inhibitors, and the protein concentration was quantified using a BCA protein assay kit (Biyuntian). Each group of proteins was denatured by boiling, and then sodium dodecyl sulfate-polyacrylamide gel electrophoresis (SDS-PAGE) was used to separate the proteins. Subsequently, the proteins were transferred to a polyvinylidene fluoride (PVDF) membrane (Millipore, USA) by electrophoresis. Then, the membranes were blocked in 5% skim milk for 1 h and incubated overnight at 4 °C in diluted primary antibody. Finally, images of the target strip were developed using a Western Chemiluminescent HRP Substrate Kit (Millipore, USA). Image Lab software (Bio-Rad, USA) was used for semi-quantitative analysis. Primary antibodies directed against AMPK (D5A2 for monoclonal antibody, Cell Signaling Technology), p-AMPK (40H9 for monoclonal antibody, Cell Signaling Technology), β-catenin (polyclonal antibodies, Cell Signaling Technology), p-β-catenin (polyclonal antibodies, Cell Signaling Technology), p-Smad-1/5/8(D5B10 for monoclonal antibody, Cell Signaling Technology), Smad-1/5/8 (N-18 for monoclonal antibody, Santa Cruz Biotechnology) and GADPH (6C5 for monoclonal antibody, Beyotime) were used.

### Transient transfection with small interfering RNAs

For RNA interference against AMPKα1/2 and CTNNB1, siRNA duplexes were synthesized, corresponding to AMPKα1/2 (target sequence: 5′-AAGAGAAGCAGAAGCACGACG-3′), CTNNB1 (sense: 5′-AUCACAGAUGUUGAAACAUTT-3′ and antisense: 5′-AUGUUUCAACAUCUGUGAUGG-3′) and control (5′-AAGCCGGTATGCCGGTTAAGT-3′). The cells were transfected with 25 nM siRNA using Lipofectamine 2000 transfection reagent (Invitrogen, USA) according to the manufacturer’s instructions and subsequently treated at 2 days after transfection.

### Immunofluorescence staining

Cells cultured in 24-well plates were fixed with 4% paraformaldehyde for 20 min at room temperature and then permeabilized using 0.5% Triton X-100 for 20 min. The fixed cells were then blocked in 5% BSA for 2 h and incubated overnight at 4 °C in a suitably diluted primary antibody against p-Smad1/5/8 (1:500, Cell Signaling). After washing with PBS, the cells were incubated with Alexa Fluor 647-labelled goat anti-rabbit IgG (H+L) antibody (1:500 dilution; Beyotime, China) at room temperature in the dark for 1 h. The cells were then incubated with DAPI for 10 min and sealed with sealing liquid containing an anti-fluorescence quencher, and images were captured on a Leica DMI 3000B inverted fluorescence microscope.

### Foetal limb explant culture

Foetal limbs were obtained by dissecting mouse embryos (E18.5) under sterile conditions and culturing in DMEM containing 0.5% BSA, 50 μg/ml ascorbic acid, 1 mMβ-glycerophosphate and 100 mg/ml penicillin and streptomycin (Sigma) at 37 °C in 5% CO_2_. After 24 h of in vitro culture, the limbs were treated accordingly. The medium was changed every 3 days. After 10 days of culture, calcein (100 mM, Sigma, USA) was added to the medium. After 12 days, the skin and muscle were removed, and new bone formation was assessed using fluorescence microscopy and histology. Each group includes at least five limb explants.

### C3H10T1/2 implantation and micro-computed tomographic (μCT) analysis

Male athymic nude mice (4 weeks old) were obtained from Shanghai Laboratory Animal Research Center (Shanghai, China). C3H10T1/2 cells were treated with AdBMP9, AdGFP and melatonin alone or in combination, and the infection efficiency was confirmed by fluorescence microscopy at 24 h later. After 7 days of treatment, the cells were harvested for subcutaneous injection (5 × 10^6^ cells per injection) into the flanks of athymic nude (nu/nu) mice (5 animals per group). Then, intrathecal injection was performed with melatonin (5 mg/kg/day) or PBS, and after 5 weeks, the animals were euthanized, and the specimen was removed and fixed. Scanning and histological evaluation of ectopic bone were performed using a high-resolution μCT system (SkyScan, Belgium) and analysis of the data using μCT analyser software (SkyScan, Belgium). All experiments were performed in accordance with the guidelines for animal experiments of the Xinhua Hospital Ethics Committee.

### Histological evaluation

The removed tissues were fixed with 4% paraformaldehyde, then decalcified and dehydrated with paraffin embedded. Serial sections of paraffin-embedded samples were stained with haematoxylin and eosin (H&E) or Masson’s trichrome stain as previously described [26, 27].

### RNA extraction and real-time reverse transcription polymerase chain reaction (real-time RT-PCR) analysis

The cells were seeded in 6-well plates, and treated with AdBMP9, AdGFP and melatonin alone or in combination for 2 days to extract total cellular RNA and perform reverse transcription. The qPCR reaction was carried out using a SYBR Green Premix Ex TaqTM kit (Takara, Japan). The reaction conditions were as follows: 95 °C for 5 s; 60 °C for 30 s; 40 cycles. GAPDH was used as the internal reference gene, and the relative quantification was performed by the ΔΔCt method (Comparative Delta-delta Ct).

### Isolation of mouse bone marrow mesenchymal stem cells (BMSCs)

Primary MSCs were collected from the bone marrow of C57 mice using the method of Soleimani et al. [28]. The 8-week-old mouse was sacrificed by cervical dislocation, and then the femur and tibia were separated. The bone marrow was washed out with Dulbecco’s modified Eagle’s medium low glucose (DMEM LG, SH30021.01, Hyclone) with a 1-ml syringe until the bones turned white. The bone marrow extract was filtered through a 70-μm cell strainer and pelleted at 1000 rpm for 5 min. The cell pellet was resuspended in 15 mL DMEM LG containing 10% FBS and 100 mg/ml Pen-Strep and inoculated into a T-75 cm^2^ flask. The solution was changed every 3 days. After the cell fusion degree reaches 80–90%, the cells are passaged, and the fourth generation can be used for experiments.

### Statistical analysis

All data are expressed as the means ± standard error (SE). The experiments were performed at least three times to ensure reproducibility. Statistical differences between the two groups were determined using one-way analysis of variance (ANOVA) or Student’s *t* test. A *P* value < 0.05 (*) was considered statistically significant.

## Results

### Melatonin synergizes with BMP9 to induce the ALP activity of C3H10T1/2 cells

**Fig. 1:**
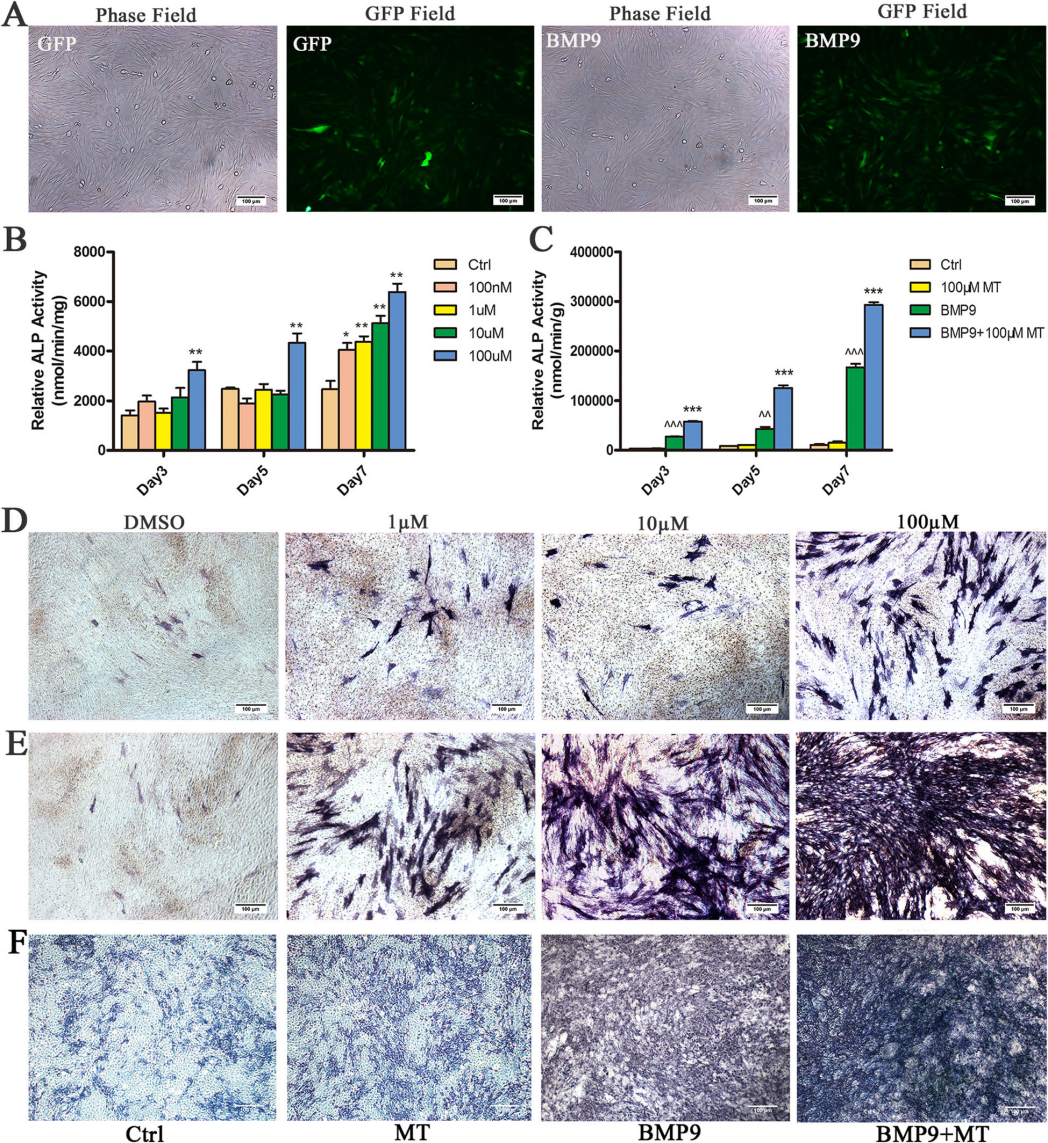
Melatonin enhances BMP9-induced early osteogenic marker alkaline phosphatase (ALP) activity in C3H10T1/2 cells. **a** AdBMP9 and AdGFP are effective in infecting C3H10T1/2 cells. **b** Melatonin induces ALP activity in C3H10T1/2 cells. **c** Melatonin cooperates with BMP9 to induce ALP activity in C3H10T1/2 cells. **d** C3H10T1/2 cells were treated with different concentrations of melatonin. **e** C3H10T1/2 cells were treated with melatonin (100 μM), AdBMP9 alone or melatonin combined with AdBMP9. **f** Primary MSCs were treated with melatonin (100 μM), AdBMP9 alone or melatonin combined with AdBMP9. Compared with the control group, ^^*p* < 0.01 and ^^^*p* < 0.001; compared with the BMP9 group, *p < 0.05 and **p < 0.01

To explore the effects of melatonin and BMP9 in synergistically inducing the osteogenic differentiation of MSC/C3H10T1/2 s, we used a recombinant adenovirus expressing human BMP9 as described above and demonstrated that this recombinant adenovirus is capable of efficiently transducing C3H10T1/2 cells (Fig. 1a). We treated C3H10T1/2 cells with 0 nM (control), 100 nM, 1 μM, 10 μM and 100 μM melatonin for 3, 5 and 7 days to determine the effect of melatonin on the osteogenic differentiation of C3H10T1/2 cells. The ALP activity of C3H10T1/2 cells increased with increasing melatonin dose and 100 μM melatonin was able to induce ALP activity to the greatest extent (Fig. 1b, d), which was selected for subsequent experiments. Next, we used melatonin (100 μM) and BMP9 alone or in combination to stimulate C3H10T1/2 cells. The ALP activity assay showed that BMP9 induced ALP activity earlier and stronger than melatonin stimulation. The combination of both melatonin and BMP9 can further enhance ALP activity (Fig. 1c, e). In addition, we performed the same experiment in primary MSC cells, and the results were similar to those in C3H10T1/2 cells, and the combination of melatonin and BMP9 further enhanced ALP activity (Fig. 1f). In summary, the data obtained indicate that melatonin can synergize with BMP9 to induce ALP activity in C3H10T1/2 cells.

### The combination of melatonin and BMP9 enhances the late osteogenic marker expression and matrix mineralization of C3H10T1/2 cells

**Fig. 2:**
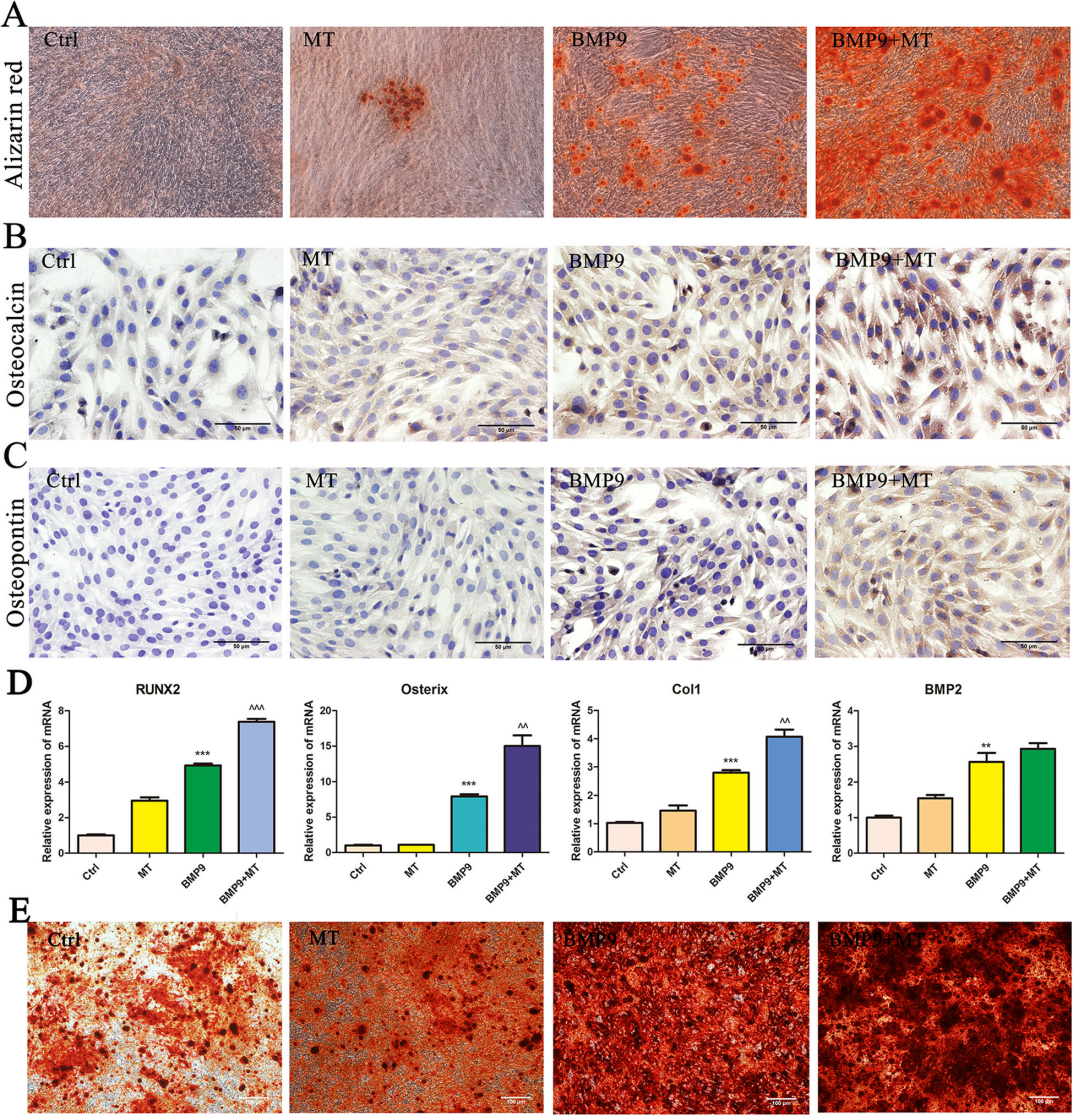
Melatonin enhances BMP9-inducedOPN and OCN expression and matrix mineralization in C3H10T1/2. **a** Alizarin red S staining. **b**, **c** Immunohistochemical staining of osteocalcin (OCN) or osteopontin (OPN). **d** The expression of osteogenesis-related genes RUNX2, Osterix, Col1 and BMP2 mRNA. **e** Alizarin red S staining of primary MSCs. Compared with the control group, ^^*p* < 0.01 and ^^^*p* < 0.001; compared with the BMP9 group, *p < 0.05 and **p < 0.01

To further confirm these results, we analysed the effect of melatonin on BMP9-induced late osteogenic markers. The results of Alizarin red S staining showed that the formation of calcium mineral deposits in C3H10T1/2 cells stimulated by melatonin and BMP9 was significantly increased compared with BMP9 infection alone, and the matrix mineralization was obvious (Fig. 2a). Immunohistochemical staining showed that melatonin significantly enhanced the expression of the BMP9-induced late osteogenic markers osteocalcin (Fig. 2b) and osteopontin (Fig. 2c). In addition, we examined the expression of osteogenesis-related genes RUNX2, Osterix, Col1 and BMP2 mRNA. The results showed that melatonin combined with BMP9 significantly increased the expression levels of RUNX2, Osterix and Col1 mRNA, while the increase of BMP2 mRNA expression levels was not statistically significant (Fig. 2d). In addition, we also performed Alizarin red S staining in primary MSC cells, and the results were similar to those in C3H10T1/2 cells, the combination of melatonin and BMP9 further enhanced the formation of calcium mineral deposits in MSCs (Fig. 2e). Based on these results, we conclude that melatonin signalling can synergize with BMP9-induced osteogenic signalling in C3H10T1/2 cells.

### Melatonin promotes BMP9-induced osteogenesis of embryonic limbs in vitro

**Fig. 3:**
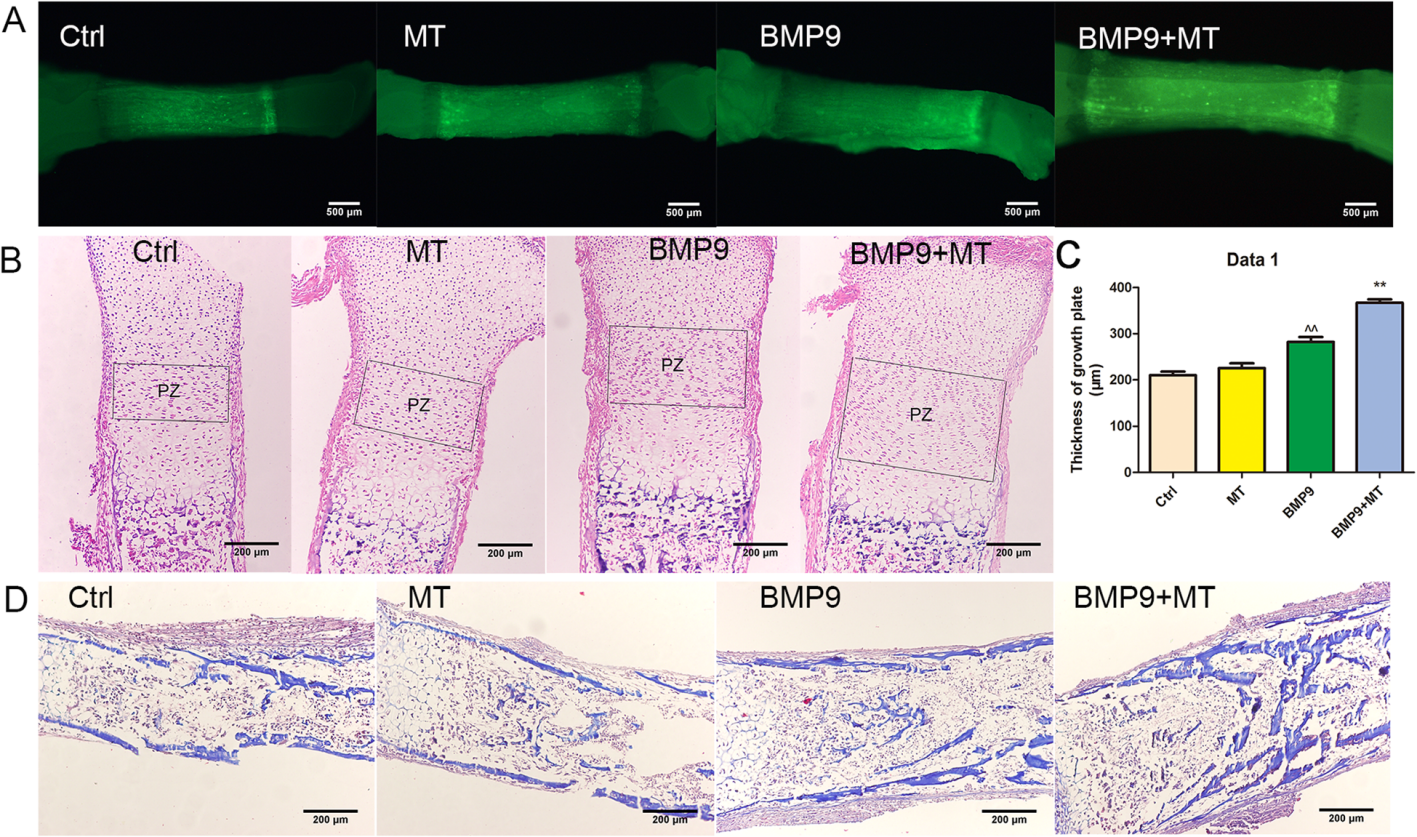
Melatonin promotes the expansion of proliferation chondrocyte regions induced by BMP9 in vitro in foetal limbs. **a** Gross image of foetal mouse tibia under fluorescence microscope. **b** H&E staining of foetal mouse tibia. The growth plates are indicated by boxes. PZ, proliferation zone. **c** Thickness of growth plate. **d** Masson’s trichrome staining of foetal mouse tibia

Next, we examined the effects of melatonin and BMP9 on bone development through foetal limb culture. E18.5 mouse embryo limbs (*n* = 5 per group) were obtained, and then the isolated limbs were infected with AdBMP9 or AdGFP in the presence or absence of melatonin (100 μM). After 10 days, the fluorescent dye calcein was added to the medium to mark new bone formation. We found that the BMP9 group and melatonin + BMP9 group were more active in bone composition and showed faster bone development than did the control group. Relative to the BMP9 group alone, the melatonin + BMP9 group showed higher fluorescence intensity, indicating that new bone formation was more active (Fig. 3a). Next, we further confirmed the above results through histological analysis. Compared with the GFP control group, melatonin or BMP9 stimulation alone increased the area of the proliferation zone, while co-treatment further increased the proliferation zone (Fig. 3b, c). Compared with the groups treated with melatonin or BMP9 alone, the melatonin + BMP9 group had thicker trabecular matrix (Fig. 3d). These results indicate that melatonin can increase the BMP9-induced endochondral ossification of foetal limb culture.

### Melatonin enhances BMP9-induced ectopic bone formation

**Fig. 4:**
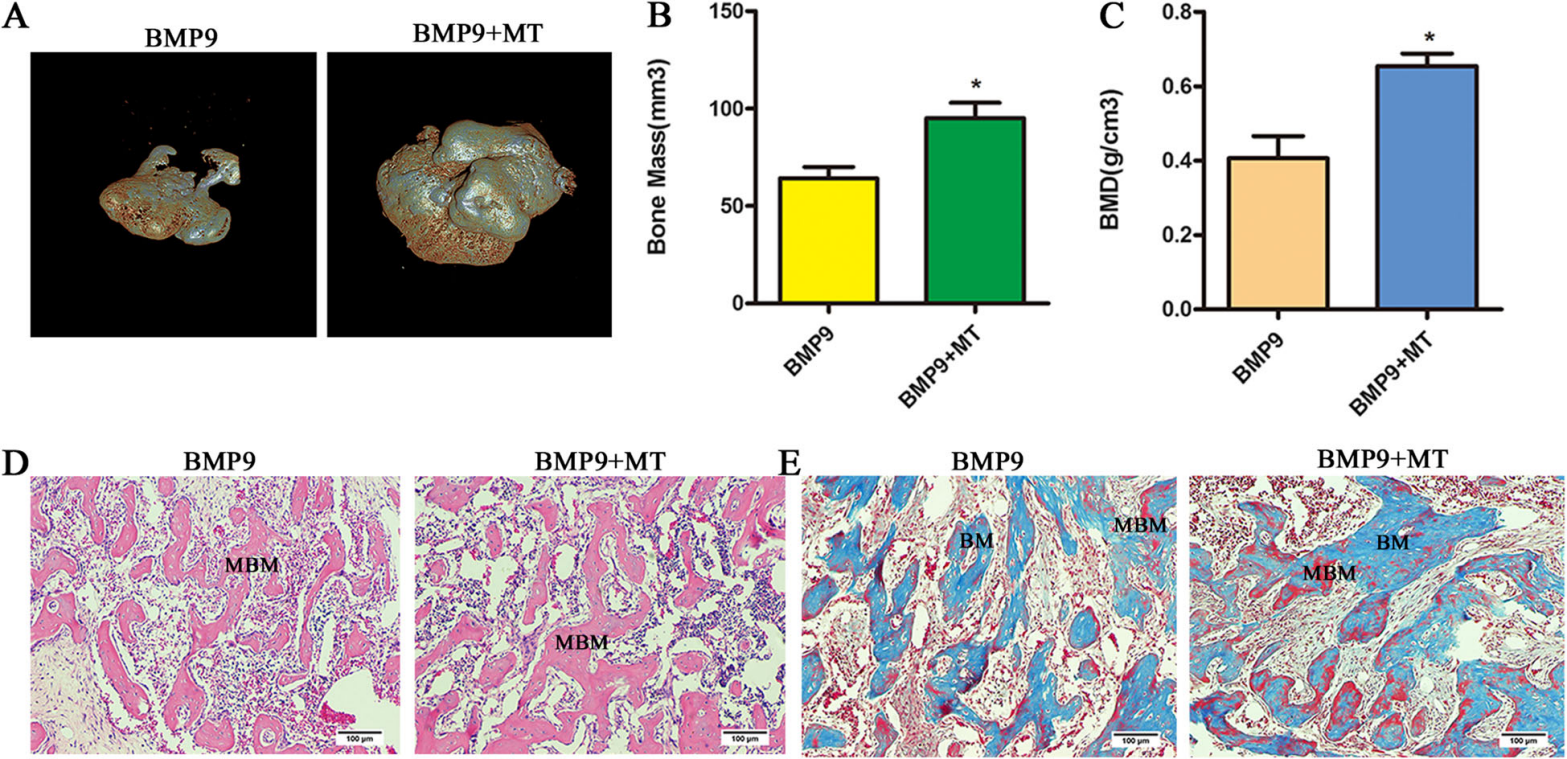
Melatonin enhances BMP9-induced ectopic bone formation. **a** Scanned image of ectopic bone using micro-CT. **b**, **c** Analysis of bone mass and relative bone density. *p < 0.05. **d**, **e** Histological analysis of specimens (H&E staining and Masson’s trichrome staining). BM, bone matrix; MBM, mineralized bone matrix

To further confirm the role of melatonin in the BMP9-induced osteogenic differentiation of C3H10T1/2 cells, we performed in vivo stem cell transplantation. We pretreated C3H10T1/2 cells with melatonin and/or BMP9 for 7 days, and the cells were harvested and injected subcutaneously into athymic nude mice, and the mice were treated with melatonin or saline subcutaneous injection. At week 6, the BMP9 group and the melatonin/BMP9 group formed ectopic bone, while the control group and the melatonin group did not have bone formation. Micro-CT scan data showed that melatonin/BMP9 treatment resulted in increased bone mass and relative bone density compared to the BMP9 group (Fig. 4b, c). The melatonin/BMP9 group formed a more mature and thicker trabecular matrix (Fig. 4d). Masson’s trichrome staining showed that melatonin enhanced the BMP9-induced mineralized bone matrix (Fig. 4e). Taken together, these data indicate that melatonin increases the BMP9-induced osteogenic differentiation of C3H10T1/2 cells.

### Melatonin promotes the BMP9-induced activation of its downstream signalling pathways in regulating the osteogenic differentiation of C3H10T1/2 cells

**Fig. 5:**
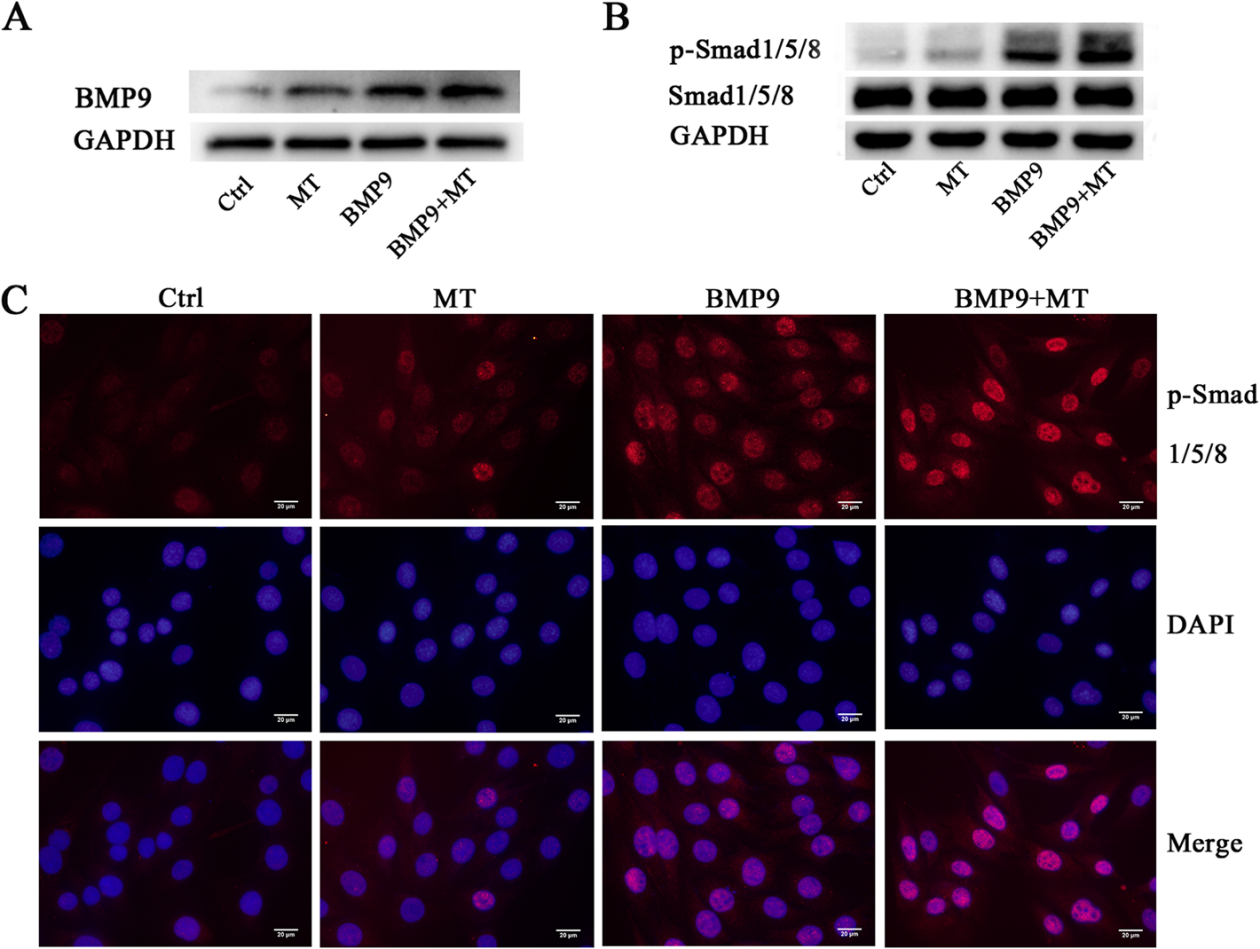
Melatonin enhances the BMP9 signalling pathway in the regulation of C3H10T1/2 osteogenic differentiation. **a**, **b** Melatonin enhances BMP9 expression and the phosphorylation of its downstream protein Smad1/5/8 in C3H10T1/2 cells. **c** p-Smad1/5/8 is transferred from the cytoplasm to the nucleus

The BMP signalling pathway plays a key role in the osteogenic differentiation of MSCs. BMP9 is considered to be one of the most potent factors in inducing the osteogenic differentiation of MSCs [4]. Studies have shown that melatonin can induce the osteogenic differentiation of MSCs and increase the expression of BMP4 and BMP6 in other cells [22, 23]. The results of our previous experiments show that melatonin can significantly enhance the osteogenic differentiation of BMP9-induced MSCs, and then we tried to determine the mechanism of interaction between melatonin and BMP9 signalling in MSCs. We found that compared to BMP9 alone, the combination of melatonin and BMP9 resulted in a significant increase in BMP9 expression and the phosphorylation of its downstream protein Smadl/5/8 in C3H10T1/2 cells (Fig. 5a, b). However, Smadl/5/8 total protein levels did not change significantly between groups. The results of immunofluorescence further confirmed that melatonin and BMP9 co-treatment significantly upregulated Smad1/5/8 phosphorylation and promoted the localization of phosphorylated Smad1/5/8 to the nucleus (Fig. 5c). These data indicate that melatonin may at least partially promote the BMP9-induced activation of BMP9/Smadl/5/8 signalling in C3H10T1/2 cells.

### Melatonin synergizes with BMP9 to promote the osteogenic differentiation of C3H10T1/2 cells via the AMPK/β-catenin pathway

**Fig. 6:**
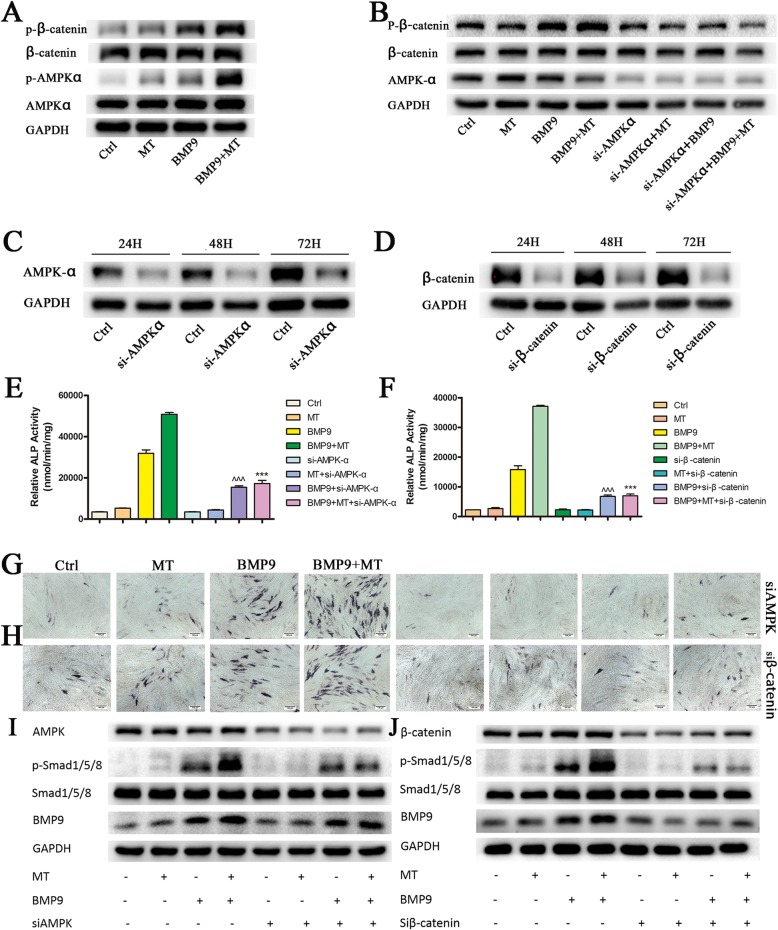
Melatonin works synergistically with BMP9 to promote osteogenic differentiation of C3H10T1/2 cells via the AMPK/β-catenin pathway. **a** Effect of melatonin combined with BMP9 on the AMPK/β-catenin pathway. **b** siAMPK inhibits the phosphorylation of β-catenin in cells co-treated with melatonin combined with BMP9. **c** siAMPK or **d** siβ-catenin has a highly potent silencing effect. **e**, **f** siAMPK or siβ-catenin abolished the ALP activity induced by melatonin combined with BMP9. Compared with the BMP9 group, ^^^*p* < 0.001; compared with the melatonin/BMP9 group, ***p < 0.001. **g**, **h** ALP histochemical staining was performed to verify the results. **i**, **j** Effect of siAMPK or siβ-catenin on the BMP9/Smad1/5/8 pathway

Studies have shown that adenosine-activated protein kinase (AMPK), an energy-sensing kinase, can regulate bone metabolism by activating beta-catenin [29]. Melatonin can protect the osteogenic differentiation function of MSCs by promoting the activation of AMPK or β-catenin [20, 30]. In multiple sources of MSCs, BMP9 and Wnt/β-catenin signalling synergistically promote osteogenic differentiation, and the knockdown of β-catenin eliminates the elevation of BMP9-induced osteogenic marker ALP [4, 12]. Therefore, we examined whether melatonin and BMP9 have an effect on the activation of AMPKα and β-catenin. Although there were no significant changes in AMPKα and β-catenin protein levels in all groups, the phosphorylation of AMPKα at Thr172 and the phosphorylation of β-catenin at Ser552 were significantly increased (Fig. 6a). To further confirm the role of AMPKα/β-catenin in melatonin- and BMP9-induced osteogenic differentiation, we treated C3H10T1/2 cells with AMPKα siRNA or β-catenin siRNA with a good silencing effect (Fig. 6c, d). Pretreatment with siAMPKα blocked the β-catenin phosphorylation induced by co-treatment with melatonin and BMP9 (Fig. 6b). Additionally, pretreatment with siAMPKα or siβ-catenin significantly inhibited the increase in ALP, phosphorylation of Smad1/5/8 and expression of BMP9 caused by the synergistic induction of melatonin and BMP9 (Fig. 6e–j). This finding suggests that the AMPK/β-catenin pathway is at least partially involved in melatonin/BMP9-induced osteogenic differentiation and that AMPK may play a role, at least in the upstream signalling of β-catenin in MSCs.

In summary, melatonin and BMP9 signalling show crosstalk between the following pathways. Melatonin/BMP9 can directly upregulate bmp9 and its downstream targets and promote the transfer of p-Smad1/5/8 from the cytoplasm to the nucleus, which is more significant than melatonin or BMP9 alone. By activating the AMPKα/β-catenin signalling pathway, melatonin/BMP9 synergizes and leads to efficient bone formation.

## Discussion

In this study, we explored the possible synergistic relationship between melatonin and BMP9 in inducing the osteogenic differentiation of mesenchymal stem cell line C3H10T1/2 cells. The results indicate that melatonin can synergize with BMP9 to induce the osteogenic differentiation of C3H10T1/2 cells through the AMPK/β-catenin pathway. Currently, the role and mechanism of melatonin on the skeletal system has not been fully clarified, and the role of the melatonin and BMP pathway interaction in the process of MSC osteogenic differentiation has rarely been reported. The mechanism of action between the two is very necessary.

BMPs play an important regulatory role in cell proliferation and differentiation during embryonic development and are key signalling pathways regulating the osteogenic differentiation of mesenchymal stem cells [7]. The interruption of BMP signalling leads to various bone and extra-bone abnormalities [31]. BMPs have the ability to regulate the multilineage-specific differentiation of MSCs. BMP2, BMP4, BMP6, BMP7 and BMP9 can effectively induce adipogenesis and osteogenic differentiation in vitro and in vivo, and BMP-induced osteogenesis or adipogenic differentiation has been shown to be mutually exclusive [12]. BMP9 is one of the most potent factors in the BMP family to induce the osteogenic differentiation of mesenchymal stem cells in vivo and in vitro, which have the ability to form bone alone. However, many signalling pathways with different functions have been found to play a role in BMP9-mediated osteogenesis, such as Wnt/β-catenin, ATRA and IGF [3, 4, 32].

Studies have shown that melatonin may have a positive effect on bone metabolism, but its osteogenesis mechanism remains unclear. Melatonin has widely distributed receptors, including G protein-coupled melatonin receptors (MT1/MT2), nuclear receptors (ROR/RZR receptors), calmodulin and mitochondria. Among these receptors, the G protein receptor (MT1/MT2) is the classical pathway for melatonin [33]. For perimenopausal women, nocturnal melatonin causes a time-dependent decrease in the ratio of serum osteoclasts and osteoblasts [34]. Similarly, as age increases, melatonin levels decrease. Bone mineral density (BMD) in the femoral neck increases after treatment with melatonin in postmenopausal women [35]. Mechanistically, although melatonin has no significant effect on the proliferation of MSCs [36, 37], melatonin has a regulatory effect on mesenchymal stem cell differentiation, which can promote osteogenic and chondrogenic differentiation [38], inhibit lipid differentiation [36] and maintain the self-renewal and differentiation characteristics of MSCs [36]. Melatonin can also play a protective role in osteogenic differentiation disorders and ageing-related osteoporosis by activating the antioxidant defence system, increasing bone formation or reducing bone resorption [13, 30, 39]. Extracellular matrix (ECM) regulates the physiological function of hormones by providing binding sites and modulating downstream signalling pathways. The interaction of melatonin with ECM deposited by natural cells can protect the osteogenic differentiation ability of MSCs [40]. Therefore, melatonin may provide a new option for the treatment of osteoporosis and fracture [19].

Previous studies have shown that melatonin promotes the expression of BMP4 or BMP6 in pituitary AtT20 cells or rat granulosa cells and the phosphorylation of Smad1/5/8 downstream of BMPs [22, 23]. Similarly, melatonin can activate osteogenic markers, such as Runx2, OCN and BMP2 and BMP4, in pre-osteoblast MC3T3-E1 cells in a dose-dependent manner [20]. Therefore, we speculate that melatonin may induce BMP9 expression during the osteogenic differentiation of C3H10T1/2 cells, and there may be a mutual promoting effect between these two factors. Our results confirm this idea, as melatonin induces BMP9 expression in C3H10T1/2 cells and enhances BMP9-induced Smad1/5/8 phosphorylation and Smad1/5/8 nuclear translocation. Although both melatonin and BMP9 were able to induce osteogenic marker expression alone, the combination of the two stimuli significantly enhanced ALP activity as well as OCN and OPN expression.

The osteogenic synthesis of collagen for bone modelling and bone remodelling requires the consumption of large amounts of adenosine triphosphate (ATP) [41]. Disorders in circadian rhythms can alter a variety of proteins that regulate glucose balance and/or energy metabolism in the body [42]. Melatonin may play a role in the regulation of energy metabolism by affecting the circadian rhythm [43, 44]. Leptin and adiponectin are adipokines that are closely related to glucose and lipid metabolism and energy balance. Circadian rhythm disorders can interfere with the synthesis and secretion of leptin and adiponectin, and melatonin supplementation normalizes the expression and secretion patterns of these two adipokines [45]. AMPK is a key regulator of energy metabolism and is regulated by a variety of factors, such as leptin, adiponectin and resistin [46, 47]. Emerging evidence suggests that AMPK regulates cell differentiation in addition to its role in metabolic processes [48, 49]: AMPK promotes osteogenesis and inhibits lipogenesis in MC3T3-E1 cells [50]; and active AMPK can directly phosphorylate RUNX2 and promote osteogenesis [51]. Therefore, we speculate that melatonin may achieve synergistic osteogenesis with BMP9 by activating AMPK. This idea is consistent with our findings that melatonin combined with BMP9 treatment increases phosphorylated AMPK levels.

Both BMP and Wnt signalling are involved in the regulation of osteoblast differentiation and bone formation. In MSCs, Wnt3a and BMP9 regulate overlapping but distinct downstream target gene expression [52, 53], suggesting that crosstalk may exist between their induced osteogenic signalling pathways, as demonstrated by subsequent studies [4, 54]. Crosstalk between AMPK and Wnt/β-catenin signalling was confirmed in both in vitro and in vivo experiments [55, 56]. AMPK phosphorylates β-catenin at Ser 552, stabilizes β-catenin and enhances β-catenin/TCF-mediated transcription, thereby regulating cell differentiation and developmental signalling pathways [46, 57]. Therefore, we propose that β-catenin may act downstream of AMPK and play a role in the osteogenic differentiation induced by melatonin in combination with BMP9. To test this hypothesis, we examined changes in AMPK/β-catenin in melatonin/BMP9-induced bone formation. The results indicate that melatonin and BMP9 have a synergistic effect on the activation of AMPK/β-catenin signalling. The knockout of AMPK or β-catenin abolishes the stimulatory effect of melatonin on BMP9-induced alkaline phosphatase activity. Therefore, we propose that melatonin combined with BMP9 may be an effective treatment for bone metabolic diseases, such as osteoporosis, and fracture healing.

However, our research also has some limitations. Studies have shown that the activation of the AMPK kinase complex induces mitochondrial division, increases apoptosis and reduces the viability of MSCs. Knocking out AMPK may help reduce MSC damage in patients with myelodysplastic syndrome [57]. Osteogenic differentiation is a multifactorial regulation process, and the molecular mechanism of the AMPK/β-catenin pathway in osteogenic differentiation of C3H10T1/2 cells induced by melatonin and BMP remains to be further studied.

## Conclusions

In summary, we investigated the effects of melatonin on BMP9-induced early and late osteogenic markers. Mechanistically, we found that this process may be at least partially mediated by the AMPK/β-catenin pathway, and silencing AMPK or β-catenin can effectively attenuate BMP9-induced osteogenesis. Therefore, the interaction between melatonin and the BMP9 pathway may play an important role in regulating the osteogenic differentiation of MSCs. Exploring the combination of melatonin and BMP9 provides a new treatment for bone metabolic diseases, such as osteoporosis.

## Data Availability

The datasets used and/or analysed during the current study are available from the corresponding author on reasonable request.

## Abbreviations

MSCs: Mesenchymal stem cells
BMP9: Bone morphogenetic protein (BMP)-9
MT: Melatonin
ALP: Alkaline phosphatase
OPN: Osteopotin
OCN: Osteocalcin
